# Intestinal Helminth Infections and Nutritional Status of Children Attending Primary Schools in Wakiso District, Central Uganda

**DOI:** 10.3390/ijerph9082910

**Published:** 2012-08-16

**Authors:** Lwanga Francis, Barbara Eva Kirunda, Christopher Garimoi Orach

**Affiliations:** 1 Department of Community Health and Behavioral Sciences, Makerere University School of Public Health, Kampala, Central Region, +256, Uganda; Email: cgorach@musph.ac.ug; 2 Department of Epidemiology and Biostatistics, Makerere University School of Public Health, Kampala, Central Region, +256, Uganda; Email: bkirunda@musph.ac.ug

**Keywords:** helminths, stunting, underweight, moderate acute malnutrition (MAM), Uganda

## Abstract

A cross-sectional study to assess the prevalence of intestinal helminth infections and nutritional status of primary school children was conducted in the Wakiso district in Central Uganda. A total of 432 primary school children aged 6–14 years were randomly selected from 23 schools. Anthropometric measurements of weight, height, MUAC were undertaken and analyzed using AnthroPlus software. Stool samples were examined using a Kato-Katz method. The prevalence of stunting, underweight and moderate acute malnutrition (MAM) was 22.5%, 5.3% and 18.5% respectively. Males had a threefold risk of being underweight (OR 3.2, 95% CI 1.17–9.4, *p =* 0.011) and 2 fold risk of suffering from MAM (OR 2.1, 95% CI 1.21–3.48, *p =* 0.004). Children aged 10–14 years had a 2.9 fold risk of stunting (OR 2.9, 95% CI 1.37–6.16, *p =* 0.002) and 1.9 risk of MAM (OR 1.9, 95% CI 1.07–3.44, *p =* 0.019). Attending urban slum schools had 1.7 fold risk of stunting (OR 1.7, 95% CI 1.03–2.75, *p =* 0.027). Rural schools presented a twofold risk of helminth infection (OR 1.95, 95% CI 1.12–3.32, *p =* 0.012). The prevalence of helminth infections was (10.9%), (3.1%), (1.9%), (0.2%) for hookworm, *Trichuria* *trichiura*, *Schistosoma* *mansoni* and *Ascaris* *lumbricoides*, respectively. The study revealed that 26.6%, 46% and 10.3% of incidences of stunting, underweight and MAM respectively were attributable to helminth infections.

## 1. Introduction

Malnutrition is an underlying cause of over half of child deaths in many developing countries and affects the physical, mental, social wellbeing and child development. Malnutrition is associated with lower enrollment and poor cognitive functioning among children attending school [1,2,3].

In Uganda, an assessment of school children aged 9–15 years in 2006–2007 revealed that 8.7%, 13% and 10.1% were stunted, underweight and thin respectively [4]. In comparison, several studies in Ghana, Tanzania, Indonesia, Vietnam and India found stunting and underweight to be high, ranging between 48% to 56% for stunting and 34% to 62% for underweight [5,6].

Globally an estimated 100 million people have been reported to have experienced stunting or wasting as a result of worm infections [7]. The nutritional status of people infected with helminths is altered through a decline in food intake and/or an increase in nutrient wastage through blood loss, vomiting or diarrhea [8]. These effects can lead to or aggravate protein energy malnutrition, anemia and other nutrient deficiencies.

In Africa, 90 million school-age children are estimated to be infected with soil transmitted helminths [9]. School-age children are more predisposed and vulnerable to helminth infections compared to other age groups [9,10,11,12]. A total of 40–50 million school-age children in sub-Saharan Africa were reported to be infected with hookworm in the year 2009 [11]. A study among 20,185 school children in Uganda revealed that 6.3%, 5.0%, 43.5% were infected with *Ascaris* *lumbricoides*, *Trichuris* *trichiura* and hookworms respectively [13].

Elimination of helminth infections can improve the nutritional status of children. The provision of periodic anti-helminth treatment in Uganda and Zanzibar has been reported to be associated with an increase in weight gain of 10% above expected [14,15]. The WHO resolved to have a 75% to 100% de-worming coverage among school-age children in countries where the prevalence of helminth infections is more than 50% [16]. In Uganda, despite the ongoing 7 year de-worming program, helminth infections remain a major health problem. The Wakiso District monthly reports of 2009 show that 23% of the 171,472 children aged below 5 years that attended the health units were infected with intestinal worms.

It is not known whether after carrying out routine de-worming for over 7 years, intestinal helminth infections still remain an important factor in the causation of malnutrition among children attending primary schools in Uganda. This study therefore, aimed at establishing whether helminth infections should be given priority as a risk factor for malnutrition among children attending primary schools in this District and Uganda in general.

## 2. Methods

### 2.1. Study Setting

The study was conducted in the Wakiso district, Central Uganda. The district has a total of 957,300 inhabitants. It is administratively made up of two counties, namely Kyandondo and Busiro, one municipality, four town councils and 15 sub-counties. In 2009 the district had a total of 774 registered primary schools 238 of which are government aided and the rest privately owned. The 774 schools had a total of 237,778 registered pupils; 118,016 (49.6%) were boys and 119,762 (50.4%) girls.

### 2.2. Sampling Procedure

This was a cross-sectional study involving 432 pupils randomly selected from 23 out of 278 schools in the selected sub-counties/town councils. Six sub-counties and two town councils were selected from the two counties. Lists of schools were drawn up and used to randomly select on average three schools from each sub-county/town council, making a total of 23 schools.

Out of the selected schools, 57% came from urban slums and 43% from rural settings. The school were stratified into private 11 (47.8%) and government 12 (50.2%). At school level the selection of the respondents was based on stratification into upper (primary 5 to 7) and lower (primary 1 to 4) respectively. One class from each stratum was randomly selected. Within the selected class, registers were obtained from which systematic sampling was done to select the children. In each stratum (class) nine or 10 pupils were randomly selected, giving a total of approximately 19 pupils investigated in each school.

### 2.3. Data Collection Procedure

Anthropometric measurements of mid-upper arm circumference (MUAC), height and weight were undertaken following Gibson’s guidelines [17] and analyzed using AnthroPlus software.

### 2.4. Weight Measurement

Children were weighed using Seca digital scales which were validated with standard weights before actual weighing of the children commenced. The scales were placed on a hard flat surface. Children wearing only lightweight clothing (excludes shoes, belts, socks, watches and jackets) were weighted. Each child was measured twice and the measure compared to agree within 10 g. If the difference between the measures exceeded the tolerance limit (the degree to which the two measurements are close), the child was repositioned and re-measured a third time. The average of the two measures in closest agreement was recorded.

### 2.5. Height Measurement

The child stood with back against the board, his/her heels, buttocks, shoulders and head touching a flat upright sliding head piece. The child’s legs were placed together with the knees and ankles brought together. Children were asked to take in a deep breath. The child’s height measurement was taken when the child had a maximum inspiration. The headpiece was brought down onto the upper most point on the head and the height recorded to the nearest 0.1 cm at the examiner’s eye level.

### 2.6. Measurement of MUAC

The MUAC was taken using the MUAC tapes recommended by WHO and UNICEF [18]. The MUAC was measured on the left arm at the level of the upper arm midpoint mark. The measurement was then taken to the nearest 0.1 cm.

### 2.7. Collection of Stool Samples

A total of 432 tool samples were collected in polyethylene containers and examined in the field using a Kato-Katz cellophane faecal thick smear method [19]. Each smear was examined twice by two different laboratory technicians. Examination of the specimen in the field was carried out in order to capture hookworms which tend to clear in the stool if not examined within a few hours of collecting the stool.

### 2.8. Quality Control

Training of research assistants and pre-testing of tools were done prior to the study. Weighing scales were checked and validated with standard weights every day before actual weighing of the children commenced.

### 2.9. Data Analysis

For anthropometric data analysis, standard deviation (Z-scores) scores were obtained by WHO AnthroPlus software [20]. Data were captured using Epi Info version 2002 and analyzed using SPSS version 11.5. Chi-squared and Fisher’s tests were used to examine differences for proportions. Odds ratios (OR) calculated by logistic regression were presented to determine risk factors for nutritional status indices.

### 2.10. Stool Analysis

Stool samples were processed and analyzed using Kato-Katz thick smear [19]. The intensity of the infection was defined by number of eggs/gram (EPG) of faeces using the World Health Organization criteria which grades the intensity as light, moderate or heavy. Light intensity infection for *T. trichiura* category was defined as 1–999 EPG and the moderate to heavy intensity infection category is defined as ≥1,000 EPG. For *ascariasis*, light-and moderate to heavy intensity infection categories were defined as 1–4,999 EPG and ≥5,000 EPG, respectively. For hookworm, light-and moderate to heavy intensity infection categories are defined as 1–1,999 EPG and ≥2,000 EPG, respectively.

### 2.11. Nutritional Status Assessment

This was assessed using BMI-for-Age (BAZ), Height-for-age (HAZ) and Mid-Upper-Arm-Circumference (MUAC). Standard deviation (SD) scores (Z scores) were applied to determine the nutritional status as recommended by the WHO [21]. Children whose BAZ and HAZ was above-2SD scores were considered well nourished and those below -2SD scores as being malnourished.

Children aged 6–10 years whose MUAC was less than 135 mm were considered to have severe acute malnutrition (SAM) while those with MUAC between 135 mm and 145 mm were considered as having moderately acute malnutrition (MAM). For children aged 10–14 years SAM was defined by a MUAC of less than 160 mm while those between 160 mm–185 mm were considered as being with MAM.

### 2.12. Ethical Considerations

Permission to carry out the study was granted by Makerere University School of Public Health Higher Degree Research and Ethics Committee and the Uganda National Council for Science and Technology (UNCST). Permission from the relevant authorities in the district was obtained before carrying out the study. Head teachers were informed in advance about the study and made arrangements for collection of stool samples. Pupils had the right to accept or refuse to join the study without any consequences. A written informed consent was sent to the parents of the pupils for endorsement. An ascent was obtained from each pupil before assessment. Children who were found to have helminth infections were treated with albendazole and praziquantel tablets.

## 3. Results

### 3.1. Socio-Demographic Characteristics of the Study Subjects

A total of 432 school children were investigated. The mean age of children was 10.9 years. The majority (78.5%) were aged between 10–14 years as shown in Table 1 below.

**Table 1:** Characteristics of children and schools.

Characteristic	n = 432	(%)
**Age group (years)**		
6–9	93	21.5
10–14	339	78.5
**Sex**		
Female	224	51.9
Male	208	48.1
**Location of school**		
Rural	203	47.0
Urban slums	229	53.0
**Ownership of school**		
Public	217	50.2
Private	215	49.8

### 3.2. Nutritional Status of Children

Table 2 shows that children in the age group of 10–14 year were 2.9 times more likely to be stunted compared to the other age group (*p =* 0.002). Children in urban slum schools were 1.7 times to be stunted compared to those rural (*p =* 0.027).

Boys were three times more likely to be underweight compared to girls (*p =* 0.011). There were no significant differences between the prevalence of underweight among children attending schools in the rural and urban slum areas (*p =* 0.438) as well as those in private and public schools (*p =* 0.128).

Table 3 shows that children in the 11–14 year age group were twice more likely to suffer from moderate acute malnutrition (MAM) as compared to those in the other age group (*p =* 0.019). Males were 2 times more likely to suffer from MAM than the girls (*p =* 0.004). There was no difference in the prevalence of MAM among children in rural and urban slum schools as well as those in public and private schools.

**Table 2:** Nutritional status of children investigated.

Variable	Nutritional Status n = 432		Odds Ratio	95% CI	*P* value
	−2SD & below n (%)	Above −2SD n (%)			
**Stunting**					
**Age (years)**					
10–14	87 (20.0)	252 (58.3)	2.9	1.37–6.16	0.002 *
6–9	10 (2.3)	83 (19.2)			
**Sex**					
Female	43 (10.0)	181 (41.9)	0.68	0.42–1.9	0.092
Male	54 (12.5)	154 (35.6)			
**Location**					
Urban slums	61 (14.2)	168 (38.9)	1.7	1.03–2.75	0.027 *
Rural	36 (8.3)	167 (38.7)			
**Ownership**					
Public	43 (10.0)	174 (40.3)	0.74	0.46–1.12	0.187
Private	54 (12.5)	161 (37.2)			
**Underweight**					
**Age (years)**					
6–9	5 (1.1)	88 (20.4)	1.0	0.32–3.00	1.0
10–14	18 (4.2)	321 (74.3)			
**Sex**					
Male	17 (3.9)	191 (44.2)	3.2	1.17–9.4	0.011 *
Female	6 (1.4)	218 (50.5)			
**Location**					
Rural	9 (2.1)	194 (44.9)	0.71	0.28–1.80	0.438
Urban slums	14 (3.2)	215 (49.8)			
**Ownership**					
Public	8 (1.8)	209 (48.4)	0.51	0.19–1.31	0.128
Private	15 (3.5)	200 (46.3)			

* p value < 0.005, statistically significant association.

**Table 3:** Nutritional status of children as measured by MUAC.

Variable	Nutritional status		Odds ratio	95% CI	*P* value
	MAM n (%)	Normal n (%)			
**Age (years)**					
11–14	60 (13.9)	215 (49.8)	1.9	1..07–3.44	0.019 *
6–10	20 (4.6)	137 (31.7)			
**Sex**					
Male	50 (11.6)	158 (36.6)	2.1	1.21–3.48	0.004 *
Female	30 (6.9)	194 (44.9)			
**Location**					
Rural	37 (8.5)	166 (38.5)	0.96	0.58–1.61	0.883
Urban slums	43 (10.0)	186 (43.0)			
**Ownership**					
Public	41 (9.5)	176 (40.7)	1.1	0.63–1.76	0.840
Private	39 (9.0)	176 (40.7)			

* p value < 0.005, statistically significant association.

### 3.3. Prevalence and Intensity of Helminth Infections

The study shows that majority of the 69 infected children had hookworms (67%) followed by *Trichiuris* *Trichiura* (19%), *Schistosoma* *Mansoni* (12%) and *Ascaris* *Lumbricoid* (2%). The intensity of infection was described as being low. The mean Egg per Gram (EPG) was 17; the lowest and highest EPG were 1 and 279 respectively. Table 4 indicates that children who attended rural primary schools were 1.95 times more likely to be infected compared to those in the urban slum schools (*p =* 0.012).

**Table 4:** Helminth infection by socio-demographic characteristics and de-worming period.

Variable	Helminth infection		Odds	95% CI	*P* value
	Infected n = 69 (%)	Uninfected n = 363 (%)	ratio		
**Age (years)**					
6–9	09 (2.1)	84(19.4)	2.01	0.92–4.54	0.061
10–14	60 (13.9)	279 (64.6)			
**Sex**					
Females	28 (6.5)	196(45.5)	0.96	0.56–1.66	0.89
Males	41(9.5)	298 (69.0)			
**Location**					
Rural	42 (9.7)	161 (37.3)	1.95	1.122–3.42	0.012*
Urban slums	27 (6.3)	202 (46.8)			
**Ownership**					
Public	36 (8.3)	181(41.9)	1.1	0.64–1.89	0.72
Private	33 (7.60)	182 (42.1)			
**De-worming period**					
July-September 2010	35 (8.1)	122 (28.2)	2.03	1.17–3.53	0.007*
October-December 2010	34 7.9)	241 (55.8)			

* p value < 0.005, statistically significant association.

**Table 5:** Comparisons of nutritional status and helminth infections.

Helminth infections	Nutritional status	Row total	Row %	95% CI	*P* value	Percentage attribution risk	Population attribution risk
	**HAZ**						
	Stunted						
	Normal						
Infected	20 49	69	29	0.8-2.8	0.2	26.6	5.8
Uninfected	77 286	363	21				
	**BAZ**						
	Underweight						
	Normal						
Infected	6 63	69	8.7	0.7-5.5	0.2	46	79
Uninfected	17	363	5				
	346						
	**MUAC**						
	MAM						
	Normal						
Infected	14 55	69	20.3	0.6-2.3	0.7	10.3	19.1
Uninfected	66 297	363	18.2				

Children who were last de-wormed between July and September 2010 had an increased risk of being infected with helminths compared to those last de-wormed between October and December 2010 (*p =* 0.007). Table 5 above indicates that 26.6%, 46% and 10.3% of the risks to stunting, underweight and MAM respectively may be due to intestinal helminth infections.

It is also important to note from Table 5 that 5.8%, 79% and 19.1% of the incidences of stunting, underweight and MAM respectively, in the total population of primary school children may be attributable to intestinal helminth infections in this district.

## 4. Discussion

The study revealed that 22.5% of the children were stunted, 5.3% underweight and 18.5% had moderate acute malnutrition. The prevalence of helminth was 16% with a low intensity of infection. The prevalence of stunting revealed by our study is lower than that reported among school children in Ethiopia of 26.5%; China; 25.6% and India 37% [22,23,24]. One explanation for the lower prevalence of stunting in this community could be the lower helminth infection prevalence and intensity of infection compared to settings where both helminth and stunting levels are high. Moderate-to-heavy intensity helminth infections have been reported to be a risk factor for the high stunting prevalence among school children [23]. A study in Nepal also revealed that out of the 818 primary school children assessed, 65.8% were infected with helminths and 61% were malnourished [25].

The prevalence of stunting was found to be significantly higher among children attending urban slum schools (14.1%) than among those in rural schools. A study by Amuta *et al.* also revealed a higher prevalence of malnutrition among children attending schools in urban slum settings of Nigeria [26]. It is possible that food insecurity may be one of the contributing factors. The socio-economic conditions of the parents/families in urban slum may be poorer than for the population in rural areas who may have agricultural fields and therefore have better access to and food security.

The study showed that the prevalence of underweight was low compared to that reported in other countries. Several studies by Medhi *et al.*, Osei *et al.*, and Nitish *et al.* done in Indian school children showed an underweight prevalence of 51.7%, 60.9% and 44%, respectively [5,24,27]. Fitsum *et al.* also reported a high prevalence of underweight of 58.3% among school-age girls in Ethiopia [22]. The low prevalence of underweight reported in our study may be attributed to the routine de-worming programme that has been running in the country since 2003. A number of studies reveal that de-worming can lead to increase in weight among those de-wormed. Provision of periodic anti-helminth treatment as part of child health services in Uganda resulted in an increase in weight gain of 10% above expected when treatments were given twice a year and an increase of 5% when treatment was given annually [14]. A randomized double blind study on *Ascaris* infested children aged 2–12 years in Dhaka (Bangladesh) also revealed a statistically significant weight gain among children given anti-helminths than those given placebos [28].

The prevalence of Underweight and MAM was significantly higher among children aged 10–14 years compared to those aged 6–9 years. These findings agree with Del Rosso’s conclusion that nutritional status does not improve with age [1]. The extra demands that come as children grow (like increased play or walking long distances to school) create a need for energy that is much greater than that of pre-school and early-school goers. Indeed, the majority (78.5%) of the children recruited in this study were teenagers aged between 10–14 years.

The prevalence of acute malnutrition (wasting) of 18.5% established by this study is somewhat similar to that reported by Medhi *et al.* and Nitish *et al.*, of 21.2% and 21.5%, respectively, among Indian school children [5,27]. The presence of moderate acute malnutrition among school goers is an indication that severe form acute malnutrition might exist in the community among school-age children however; this study may not have been able to capture them because a severely acutely malnourished child cannot attend school.

The study revealed a significantly higher prevalence of underweight and moderate acute malnutrition (MAM) among boys than girls. Our results are consistent with those of Ingunn Marie *et al.* and Mupfasoni *et al.*, which showed that boys are more vulnerable to malnutrition compared to girls [29,30]. The biological, psychosocial and cognitive changes associated with rapid growth and development in teenagers directly affect their nutritional status and nutrient needs. These needs are however, different for boys and girls [31].

The study showed that 16% of the children were infected with intestinal helminth. The prevalence of these infections in this study is lower than that reported by Kabatereine *et al.* [13,32]. The fact that the studies by Kabatereine *et al.* were undertaken only 2–3 years after starting a country wide de-worming policy as compared to this done after 7 years might explain the lower prevalence of helminth infections revealed by this study. Thus the policy of child days plus during which de-worming of children take pace may be having some effects.

The study revealed that children who were last de-wormed more than 6 months to the time of research were twice more likely to be infected with helminths than those last de-wormed less than six months. This may suggest that helminth re-infections may be occurring frequently during the year. This may be associated with poor sanitation environment in which the children live in the urban slums and rural communities.

Children attending rural schools had a higher risk of being infected with helminth infections compared to those in the urban slums. These findings are not different from those of Stoltzus *et al.*, where hookworms were more prevalent among the rural Pemba island school children than those in the urban areas [33]. It is important to note that none of the children investigated had a moderate to heavy form of infection. All (16%) the infected children had a very light intensity of infection. It is possible that the de-worming campaign that has been running in this country since 2003 could have reduced the prevalence and intensity of the helminth infections.

The study showed that acute and chronic malnutrition is high among children attending primary schools. Urban slumming is a risk factor for chronic malnutrition while early adolescence and being a male child are a risk to acute malnutrition. Therefore programs directed towards improving the nutritional status of school children should be formulated, monitored and periodically evaluated. Such programs should treat males and teenage school goers as children more vulnerable to malnutrition and thus make special arrangements for improvement of their nutritional status.

The study revealed that 26.6%, 46% and 10.3% of the incidences of stunting, underweight and moderate acute malnutrition (MAM) cases respectively are attributable to the potential effects of intestinal helminthes infections. The study also showed that, 5.8%, 79% and 19.1% of the incidences of stunting, underweight and MAM in the total population of primary school goers in this district are attributable to intestinal helminthes infections. These findings do not differ much from those of Shang who mentioned moderate-to-heavy intensity helminthes infections a risk factor for the 25.6% stunting prevalence among school children in China [23].

## 5. Conclusions

Basing on the findings of this study, the following conclusions can be made:

1. Acute malnutrition in its moderate form and stunting is high among children attending primary schools. Living in urban slums is a risk factor for stunting while early adolescence and being a male child are a risk to moderate acute malnutrition.
2. The intensity of helminthes infections among school children in this district is very low. The low intensity being attributable to the bi-annual de-worming campaign that has been ongoing for over 7 years.
3. Elimination of helminthes infections among school children can play a major role in improvement of the children’s nutritional status.
