# Correction: Lwanga, F., *et al*. Intestinal Helminth Infections and Nutritional Status of Children Attending Primary Schools in Wakiso District, Central Uganda. *Int. J. Environ. Res. Public Health* 2012, *9*, 2910-2921

**DOI:** 10.3390/ijerph9103769

**Published:** 2012-10-23

**Authors:** Francis Lwanga, Barbara Eva Kirunda, Christopher Garimoi Orach

**Affiliations:** 1 Department of Community Health and Behavioral Sciences, Makerere University School of Public Health, Kampala, Central Region, +256, Uganda; Email: cgorach@musph.ac.ug; 2 Department of Epidemiology and Biostatistics, Makerere University School of Public Health, Kampala, Central Region, +256, Uganda; Email: bkirunda@musph.ac.ug

The author wish to make the following correction to this paper [1]: The author name “Lwanga Francis” should be “Francis Lwanga”.
